# Sirtuin 3 (SIRT3) Pathways in Age-Related Cardiovascular and Neurodegenerative Diseases

**DOI:** 10.3390/biomedicines9111574

**Published:** 2021-10-29

**Authors:** Ciprian N. Silaghi, Marius Farcaș, Alexandra M. Crăciun

**Affiliations:** Department of Molecular Sciences, University of Medicine and Pharmacy “Iuliu Hațieganu”, 400012 Cluj-Napoca, Romania; farcasmarius47@gmail.com (M.F.); acraciun@umfcluj.ro (A.M.C.)

**Keywords:** SIRT3, aging, neurodegenerative disease, cardiovascular disease, NAD^+^, acetylation

## Abstract

Age-associated cardiovascular and neurodegenerative diseases lead to high morbidity and mortality around the world. Sirtuins are vital enzymes for metabolic adaptation and provide protective effects against a wide spectrum of pathologies. Among sirtuins, mitochondrial sirtuin 3 (SIRT3) is an essential player in preserving the habitual metabolic profile. SIRT3 activity declines as a result of aging-induced changes in cellular metabolism, leading to increased susceptibility to endothelial dysfunction, hypertension, heart failure and neurodegenerative diseases. Stimulating SIRT3 activity via lifestyle, pharmacological or genetic interventions could protect against a plethora of pathologies and could improve health and lifespan. Thus, understanding how SIRT3 operates and how its protective effects could be amplified, will aid in treating age-associated diseases and ultimately, in enhancing the quality of life in elders.

## 1. Introduction

Aging is the principal risk factor for diseases prevalent in developed nations, i.e., neurodegeneration, cancer and cardiovascular diseases [1]. The function of mitochondria physiologically declines with age (due to mitochondrial DNA mutations and the decreased activity of mitochondrial enzymes) and contributes to the development of age-associated diseases [2]; therefore, maintaining mitochondrial health throughout an organism’s life is vital for cellular homeostasis and prevents the appearance of age-associated diseases [3,4,5].

Sirtuins (SIRTs) are nicotinamide adenine dinucleotide (NAD^+^)-dependent enzymes that regulate energy metabolism, mitochondrial activity and aging [6,7,8]. Among the seven homologs (SIRT1-7) identified to date, mammalian SIRTs adjust their catalytic activity to cellular NAD^+^ levels, contributing to cellular adaptations, stress response and to healthy aging and longevity [9,10]. Increased SIRTs activity delays the onset of age-associated diseases [11,12] and increases mitochondrial biogenesis in skeletal muscle as an adaptation to exercise [13]. Moreover, increasing SIRTs activity via NAD^+^ boosters can have numerous protective effects against cardiovascular disease and could extend health- and lifespan [14].

SIRT3, one of the three mitochondrial SIRTs (SIRT3, SIRT4, SIRT5), is localized in the mitochondrial matrix, where it contributes to cellular stress responses [15,16]. Mitochondrial proteomic data showed that SIRT3 regulates about 100 proteins [16,17,18] that are involved in β-oxidation, antioxidation, amino acid metabolism or mitochondrial permeability proteins [19,20].

SIRT3 decreases cellular oxidative stress, increases reactive oxygen species (ROS) clearance, enhances the electron transport chain efficacy and stimulates the glutathione production that neutralizes ROS [21,22,23]. Overall, SIRT3 modulates the activity of molecules that affect mitochondrial function, metabolism and oxidation responses [24,25], and protects against the development of age-associated diseases [15]. In addition, SIRT3—the principal mitochondrial deacetylase [17]—mainly exerts its function by regulating the acetylation of lysine residues on mitochondrial proteins [26]. Among the three SIRTs contained in the mitochondrial matrix, SIRT3 is the major regulator of organelles’ acetylome [27,28], as only SIRT3 deletion results in mitochondrial protein hyperacetylation [26].

Acetylation is a post-translational modification that involves the transfer of an acetyl moiety from acetyl-CoA to the lysine residues of a protein. Acetylation influences protein activity [29] and the local protein interactome [30], mainly exerting inhibitory effects [31]. Reversible acetylation also regulates protein function: stability, enzymatic activity, protein-protein interaction and subcellular localization [32,33,34]. SIRT3 removes acetyl moieties from the lysine residues of mitochondrial proteins involved in oxidative stress, energy metabolism and membrane permeability and, therefore, regulates their activity [17,34,35]. Deacetylation is also a key regulator of processes relevant to aging [36,37]. Most mitochondrial proteins are quickly deacetylated for metabolic and bioenergetic reprogramming during aging [36]. SIRT3 knockout mice have a hyperacetylated mitochondrial proteome and low electron transport chain subunit levels [38]. Conversely, SIRT3 activation relieves adenosine triphosphate (ATP) production [35,39] and may protect against oxidative stress [40].

As a byproduct of cell respiration that strongly increases with age [41], ROS contribute to the cumulative damage of cellular proteins and ribonucleotides that leads to apoptosis, cell-cycle arrest and aging. SIRT3 modulates cell programs that combat excessive ROS.

Here, we review the up-to-date signaling pathways and molecular mechanisms by which SIRT3 regulates the biology of aging and age-associated diseases, especially cardiovascular and neurodegenerative diseases, and how interventions that target these pathways could improve health and increase lifespan.

## 2. SIRT3 in Aging Biology

### 2.1. SIRT3, Oxidative Stress and Acetylation Status

The genetic polymorphisms that upregulate SIRT3 activity are associated with increased human longevity [42]; conversely, a SIRT3 mutation that decreases its activity is correlated with a high risk of developing metabolic syndrome [43,44,45]. SIRT3 is highly expressed in long-lived populations, with multiple lines of evidence suggesting that increased SIRT3 gene expression or increased enzymatic activity can extend the human lifespan [43,46]. It has been shown that SIRT3 gene variants with increased transcriptional activity promote longevity in humans [45]. Serum SIRT3 and transcription factor Forkhead box O3 (FOXO3A) protein levels were significantly higher in young individuals compared to the elderly [47]. Moreover, NAD^+^ levels were found to decrease as organisms aged, contributing to lower SIRT3 activity [48].

FOXO transcription factors regulate mammalian cell growth, differentiation, apoptosis and longevity [49]. FOXO3A is downregulated in multiple age-associated pathologies [50]. In mitochondria, SIRT3 interacts with FOXO3A by attaching to each other, consequently promoting manganese superoxide dismutase (MnSOD) and catalase transcription [51]. SIRT3 overexpression increases FOXO3a gene expression and the activity of its protein product and decreases cellular oxidized glutathione and superoxide levels [51]. Conversely, decreased SIRT3 activity promotes the appearance of age-associated pathologies: mice lacking SIRT3 develop diseases characteristic of aging (e.g., metabolic syndrome, liver, lung and hearth fibrosis) [52,53].

FOXO3A regulates ROS signaling by upregulating superoxide dismutase 2 (SOD2) [54]. SIRT3 synergizes with FOXO3A in promoting mitochondrial DNA (mtDNA) RNA polymerase activity and upregulates the expression of all mitochondrial genes. SIRT3 decreases the mitochondrial membrane potential and ROS production and increases cell respiration [22].

In mice, SIRT1 regulates SIRT3’s acetylation status; a small proportion of SIRT1 is located inside the mitochondria [55,56] where it directly targets, deacetylates and activates SIRT3. Aging and obesity decrease the SIRT1-regulated deacetylation of SIRT3. Acetylated SIRT3 is targeted for degradation and has reduced deacetylase activity, because the acetylation of SIRT3 inhibits its deacetylase activity [19]. SIRT1 deacetylates and induces peroxisome proliferator-activated receptor (PPARα) and peroxisome proliferator-activated receptor-gamma coactivator (PGC-1α) expression, which, in turn, increase SIRT3 gene expression [44,57,58]. In the hepatic tissue of SIRT1 liver knock-out (KO) mice, the PGC-1α and SIRT3 messenger RNA (mRNA) levels were decreased. SIRT1 increased SIRT3 levels by increasing its stability and the expression of its gene. Decreased NAD^+^ levels and SIRT1 activity may contribute to SIRT3 hyperacetylation and increased mitochondrial protein acetylation [44]. Therefore, acetylated SIRT3 may be a potential therapeutic target in age-related metabolic disorders.

SIRT3 KO mice have significantly shortened lifespans [59]. SIRT3 deficiency leads to defective mitochondrial oxidative phosphorylation, promoting decreased ATP synthesis and increased ROS [22]. Conversely, in aged and obese mice, mitochondrial SIRT3 levels decrease and manifest higher levels of acetylation compared to those present in their younger and leaner counterparts [60,61]. SIRT3 expression and enzymatic activity are reduced in aging and obesity [25,44,62]. Additionally, NAD^+^ levels are also decreased in those conditions, further contributing to decreased SIRT activity [63,64].

One of the principal mechanisms by which SIRT3 activation hinders disease progression is the deacetylation and activation of key enzymes that combat oxidative damage. In this respect, in in vitro fibroblasts, it has been proven that SIRT3 deacetylates FOXO1, which increases the expression of FOXO1-targeted genes (MnSOD, catalase) and decreases the senescence induced by high glucose conditions [65]. By targeting this specific SIRT3 pathway, mitochondrial dysfunction and excessive ROS may no longer be ubiquitous accelerators of age-associated diseases.

### 2.2. SIRT3 and Visceral Fibrosis

Fibrosis is a hallmark of aging [52] and, in terms of a molecular mechanism, is developed via myofibroblasts that synthesize excessive levels of extracellular matrix, cytokines, inflammatory molecules and growth factors that promote fibrosis [66]. Due to aging and chronic disease, myofibroblasts multiply and persist in the tissues, leading to fibrosis that culminates with organ failure [67]. Transforming growth factor β (TGF-β) signaling is among the principal fibrosis contributors via binding membrane receptors that lead to the activation of Smad transcription factors (Sma and Mad proteins from *Caenorhabditis elegans* and *Drosophila*, respectively), transforming fibroblasts into myofibroblasts, which finally generate an extracellular matrix. Aging and chronic tissue insults accelerate TGF-β signaling, increasing fibrosis [52,68], while caloric restriction suppresses it [69].

SIRT3 deficiency induces TGF-β synthesis and promotes the expression of transcription factors that activate pro-fibrotic genes (NFATc and beta-catenin) [53]. SIRT3 does not reduce fibrosis by decreasing ROS levels or by binding to the promoters of fibrotic genes. SIRT3 KO mice show higher visceral fibrosis with aging and increased blood pressure compared to wild-type controls. In addition, SIRT3 deficiency induces human cardiac fibroblast-to-myofibroblast differentiation, as SIRT3-depleted human cardiac fibroblasts showcased an increased myofibroblast marker expression. Moreover, fibroblasts from end-stage heart failure patients showed reduced SIRT3 and high myofibroblast marker levels, suggesting that SIRT3 deficiency induces age-dependent visceral fibrosis by promoting fibroblast-to-myoblast differentiation. SIRT3 negatively regulates the TGF-β promoter and downregulates its synthesis, achieving the endpoint indirectly via the negative regulation of c-Jun, a nuclear transcription factor, since SIRT3 does not bind directly to the TGF-β gene promoter [53].

Optic atrophy 1 (OPA1), a fusion-promoting protein of the inner mitochondrial membrane, was found to be reduced and hyperacetylated in SIRT3 KO mice and, moreover, restoring deacetylated OPA1 was found to normalize mitochondrial cristae and ATP levels [59]. SIRT3^−/−^ mice showcased reduced mitochondrial complex one activity and decreased ATP and NAD^+^ production. SIRT3 deacetylates OPA1, which preserves normal mitochondrial cristae architecture and mitochondrial function [59,70]. The deacetylated, active form of OPA1 corrects cardiac fibrosis and malfunction, OPA1 deacetylation via SIRT3 being the only known post-translational change that activates OPA1.

Glycogen synthase kinase 3 beta (GSK3β), a kinase that regulates aging and tissue fibrosis [71], is regulated via reversible lysine acetylation. Acetylation negatively regulates its kinase activity. GSK3β undergoes intra-mitochondrial deacetylation. Then, deacetylated GSK3β is most likely relocated to the cytosol, where it exerts its function [53]. SIRT3 binds to and deacetylates GSK3β, consequently increasing its kinase activity, which inhibits pro-fibrotic gene expression (beta catenin, Smad3, c-Jun) and decreases age-associated fibrosis [53].

The presented findings support the hypothesis that SIRT3 activation prevents age-associated visceral fibrosis via different signaling pathways (e.g., TGF-β, OPA1 and GSK3β).

Recently published data validated the role of SIRT3 as a potential fibrosis inhibitor. Aging is associated with a decreased regenerative capacity and contributes to fibrotic disorders in humans. SIRT3 is downregulated in the pulmonary tissue of mice with lung damage and also in individuals with idiopathic pulmonary fibrosis [72]. SIRT3 overexpression improved the ability of aged mice to decrease fibrosis levels. Thus, SIRT3 can influence macrophage function in a positive manner, leading to fibroblast apoptosis and the resolution of fibrosis [72].

## 3. SIRT3 in Cardiovascular Diseases

Cardiovascular diseases (CVD) are a leading cause of morbidity and mortality in the elderly population [73]. Increased SIRT3 activity is associated with an increased healthspan and lifespan in humans [45,74], which is supported by clinical data indicating that SIRT3 decreases with age and CVD risk factors [75,76]. Mitochondrial malfunction has a causal role in CVD, as the heart is the organ with the highest mitochondrial density and oxygen consumption; hence, SIRT3 deficiency strongly affects its function [77].

SIRT3 KO mice have a reduced cardiac mitochondrial mass, dysfunctional mitochondrial networks and Ca^2+^ homeostasis, impaired cardiac contraction, reduced ATP synthesis and abnormal cardiac mitochondrial cristae architecture [59]. Those conditions compromise mitochondrial respiration [78], reduce lifespan and lead to the development of age-associated cardiac pathology [59]. Conversely, SIRT3 activity protects against doxorubicin-induced cardiomyopathy [79] and against mitochondrial-induced apoptosis [80] by deacetylating and activating OPA1, thus maintaining a mitochondrial cristae structure and preventing cytochrome C diffusion in the cytosol [81,82,83].

SIRT3 KO human cardiomyocytes have altered mitochondrial clustering, showing fewer and morphologically altered perinuclear mitochondria [84,85,86]. Therefore, mitochondrial clustering is important for ATP generation and mitochondrial–nucleus energy communication. Furthermore, mitochondrial dysfunction in cardiomyocytes leads to impaired bioenergetics and decreased cell survival, consequently contributing to age-associated cardiac fibrosis [87,88,89].

### 3.1. SIRT3 Modulates Endothelial Dysfunction, Hypertension and Atherosclerosis

SIRT3 protein and mRNA levels are decreased in aged rats and human veins; however, a SIRT3 gene transfer increased the following cellular components: SIRT3 expression and concentration, catalase, nitric oxide (NO), MnSOD and improved vascular parameters [90]. SIRT3 deficiency leads to endothelial dysfunction and insulin resistance in humans and rodents with obesity [56]. With age, defective mitochondria accumulate and mitochondrial biogenesis decreases [91]. Excessive mitochondrial ROS (mtROS) leads to mitochondrial DNA damage, respiratory chain dysfunction, endothelial inflammation, atherogenesis and HT [92,93]. Moreover, antioxidants such as vitamin E and ascorbate do not prevent HT or CVD and no drug approved to date targets mtROS. Thus, targeting the expression level or the stability of enzymes that scavenge excess free radicals could constitute a paradigm shift in decreasing oxidative damage.

In endothelial cells, hypoxia increases SIRT3 expression, which deacetylates, stabilizes and decreases FOXO3 degradation. These mechanisms increase the FOXO3-dependent upregulation of antioxidant enzymes (e.g., peroxiredoxin 3, thioredoxin 2, MnSOD) and mtROS clearance, thus preserving mitochondrial function and improving cell survival in hypoxic states, and ultimately deterring CVD development [94].

Human umbilical vein endothelial cells exposed in vitro to high glucose concentrations showcase enhanced apoptosis, suppressed mitochondrial respiratory complexes one and two, decreased ATP production, reduced mitochondrial membrane potential and increased ROS induced by a decrease in antioxidant enzymes (e.g., glutathione, SOD and glutathione peroxidase) [95]. Notably, SIRT3 reverses mitochondrial dysfunction, normalizes ATP content, decreases cell death (SIRT3 inhibits caspase-3 and Bad activation and increases Survinin and x-IAP levels), impairs ROS generation (by preventing decreases in reduced glutathione, SOD and glutathione peroxidase), represses malondialdehyde and normalizes mitochondrial membrane potential [95].

**Figure 1:**
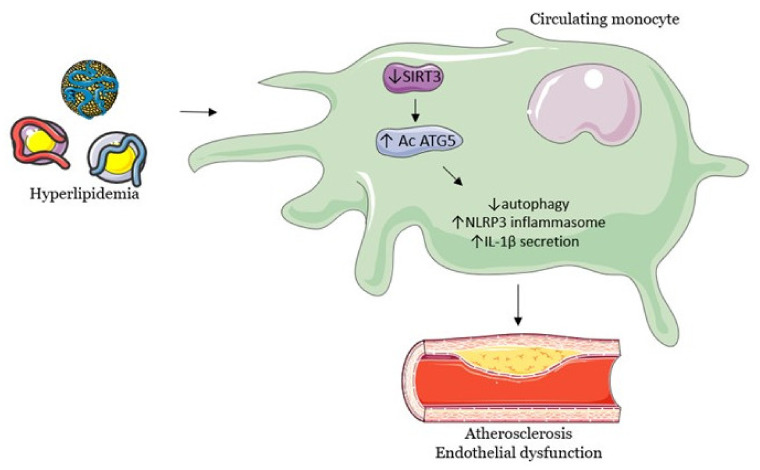
Hyperlipidemia leads macrophages to induce endothelial dysfunction and promotes atherogenesis. Abbreviations: SIRT3, sirtuin3; Ac ATG5, acetylation of autophagy-related protein 5; NLRP3, NOD-, LRR- and pyrin do-main-containing protein 3; IL-1β, interleukin-1β.

On the same note, in vitro hyperlipidemia inhibits monocytic SIRT3, which leads to autophagy, mitophagy and decreased levels of endothelial adhesion molecules, thus contributing to endothelial dysfunction, inflammation and atherogenesis [96]. The proposed mechanism is that the loss of SIRT3 activity promotes the hyperacetylation of autophagy-related protein 5 and the activation of NLRP3 inflammasome (NOD-, LRR- and pyrin domain-containing protein 3), with a high release of interleukin-1β, which supports the increased expression of endothelial adhesion molecules and atherogenesis. The same study found that a loss of SIRT3 activity in mice also promotes hyperlipidemia, inflammation and endothelial dysfunction [96]. The pathway by which decreased SIRT3 levels lead to a cascade of changes relevant to the cardiovascular system, ultimately promoting the development of atherosclerosis, is summarized in Figure 1.

HT is a primary risk factor for age-associated diseases [97,98]. HT predisposes humans and rodents to neurodegeneration, cognitive deficiency and dementia (i.e., HT-associated dysfunction in hippocampal synaptic plasticity and hippocampal synaptic loss, leading to cognitive deficiencies, aging-like phenotypes and accentuated Alzheimer’s symptomatology) [99,100,101,102]. In addition, cigarette smoke, a validated risk factor for CVD, decreases endothelial SIRT3 levels and leads to SOD2 hyperacetylation and endothelial dysfunction [103].

In hypertensive mouse models with defunct mitochondria, the administration of a mitochondrial-targeted SOD2 mimetic decreases blood pressure and improves endothelial function [104]. The proposed mechanism states that SIRT3 deacetylates and activates SOD2 [105], which protects against endothelial dysfunction and HT [106,107]. SIRT3 depletion leads to SOD2 hyperacetylation, inactivation and increased mitochondrial oxidative stress and HT [103,108]. Moreover, peripheral blood mononuclear cells from hypertensive humans show decreased SIRT3 levels and increased SOD2 acetylation, supporting the correlation between HT and SIRT3 depletion [108]. Another study that supports this mechanism found that mediastinal fat-derived arterioles from human subjects with essential HT have a threefold increase in SOD2 acetylation, a 40% decrease in vascular SIRT3 and a three to four-fold increase in cellular senescence and inflammation markers compared to those from normotensive subjects [109]. The underlying finding was that angiotensin 2 (ANGII) increases vascular hypoxia-inducible factor 1-alpha (HIF1alpha) and reduces Ve-cadherin (associated with higher vascular permeability), a process that increases the cytokine permeation in perivascular tissues, contributing to hypertrophy, inflammation, HT and viscera damage. Finally, SIRT3 overexpression prevents HIF1alpha upregulation, maintains lower Ve-cadherin levels and normalizes endothelial permeability [109].

The mounting evidence in animal models continues to reveal the beneficial role of SITR3 in modulating HT and endothelial dysfunction. In mice, SIRT3 deficiency in endothelial cells induces diastolic dysfunction and coronary artery fibrosis [110]. Moreover, in hypertensive mouse models, systemic SIRT3 overexpression decreases HT and the hypertrophy of vascular smooth muscle and prevents vascular oxidative stress (excessive vascular superoxide production and decreased endothelial NO), reaffirming the central role of SIRT3 in vascular function and HT prevention [108,109].

In addition, endothelial progenitor cells, pivotal in the re-endothelization after vascular intimal injuries, are dysfunctional in HT [111]. In endothelial progenitor cells, HT decreases SIRT3 expression and SIRT3-SOD2 signaling while increasing mtROS and oxidative damage [112]. The study also revealed that upregulating SIRT3 expression leads to SOD2 deacetylation, a reduction in oxidative damage and an increase in the re-endothelization capacity of the endothelial progenitor cells.

Compared to wild-types controls, SIRT3 KO hypertensive mice developed severe microvascular rarefication, cardiac hypoxia, mitochondrial dysfunction and impaired mitophagy due to Pink/Parkin hyperacetylation, increased collagen gene expression and cardiac fibrosis [113]. Similar to previous studies, SIRT3 overexpression reversed the aforementioned dysfunctions and improves cardiac vascularization and function. Moreover, in an ANGII hypertensive mouse model, renal SIRT3 deficiency promoted the differentiation of endothelial cells to a mesenchymal phenotype, which decreases FOXO3-dependent catalase gene expression and increases oxidative stress and renal fibrosis [114].

A different mechanism of action for SIRT3 is via an ROS-activated nuclear factor kappa-light-chain-enhancer of activated B cells (NFkB). Several studies found that ROS-activated NFkB mediated vascular inflammation and induced vascular cell adhesion molecule 1 (VCAM-1) and monocyte chemoattractant protein-1 (MCP-1) that, in turn, increased vascular malfunction and inflammation [115,116,117]. SIRT3 depletion led to NLRP3 inflammasome and NFkB activation, with SIRT3-depleted aortas showing vascular inflammation (increased VCAM-1 and MCP-1). The findings were consistent with ANGTII-induced vascular inflammation, where SIRT3 overexpression decreased vascular VCAM-1, MCP-1 and vascular inflammation, respectively [109].

There is a plethora of interventional studies on experimental models detailing the functions of SIRT3. Hydrogen peroxide induces SIRT3 to deacetylate FOXO3, which upregulates PGC-1 and mitochondrial transcription factor A expression (regulates ATP production and mitochondrial mass), thus maintaining mitochondrial homeostasis [118]. In respect to the AMP-activated protein kinase (AMPK) pathway, a sensor for cellular energy, the administration of Metformin and Nitrite activates the SIRT3-AMPK signaling pathway, consequently leading to the normalization of pulmonary hypertension and an improvement of hyperglycemia in mouse heart failure models [119]. Additional interventional studies focused on HT are presented in Table 1 [120,121,122,123].

These studies were conducted using global transgenic mice models. Future studies should examine the effects of viscera- or cell-specific SIRT3 transgenicity and the mechanism by which HT decreases SIRT3 expression. Overall, SIRT3 activators may be more efficient than antioxidants at decreasing oxidative stress and preventing CVD.

### 3.2. Cardiac Hypertrophy

There are multiple interventional studies focused on the involvement of SIRT3 pathways in cardiac hypertrophy. A summary of this research is shown in Table 2 [116,117,118,119,120,121,122,123,124,125].

SIRT3 negatively regulates hypertrophy-inducers by acting on multiple cellular compartments. In models of cardiac hypertrophy, SIRT3 mRNA, protein expression and enzymatic activity are constitutively reduced [134]. SIRT3 KO mice have cardiac inflammation, fibrosis [135], lower ATP levels and develop cardiac hypertrophy at an early age [136]. In line with a previous study, SIRT3 deficiency leads to an increased acetylation of mitochondrial metabolic proteins, which induces cardiac hypertrophy [137] and accelerates obesity-induced heart failure [138]. Moreover, SIRT3 overexpression protects cardiomyocytes from apoptosis and hypertrophy [80].

SIRT3 regulates cardiac ATP generating pathways by altering the lysine acetylation of numerous mitochondrial enzymes. In SIRT3 KO mice, over 84 cardiac mitochondrial proteins are hyperacetylated, ranging from oxidative phosphorylation to fatty acid oxidation enzymes, contributing to a decrease in their activity [137]. A decreased deacetylation of fatty acid oxidizing enzymes leads to the accumulation of lipids in the mitochondria, but the overexpression of SIRT3 inhibits cardiac hypertrophy by decreasing lipid accumulation [139]. In mice, the deletion of endothelial SIRT3 increases oxidative phosphorylation, reduces glycolysis and impairs angiogenesis, leading to diastolic dysfunction, cardiac hypertrophy and an age-related decrease in heart function [137]. In addition, SIRT3 deficiency impairs mitochondrial oxidation and endothelial function, but also decreases angiogenic growth factors and induces microvascular rarefaction, resulting in cardiac energy depletion, contractile dysfunction, heart failure and impaired recovery [137,140].

On one hand, excessive oxidative stress can decrease cellular NAD^+^ levels, resulting in an impaired SIRTs function [141]. Thus, pathologic cardiac hypertrophy is associated with decreased cellular NAD^+^ levels, and supplementation with NAD^+^ activates cardiac SIRT3 and blocks ANGII-induced hypertrophy. Moreover, enzymes that increase NAD^+^ levels may indirectly activate SIRT3. Yue et al. [134] found that SIRT3 overexpression protects myocytes against hypertrophy by deacetylating nicotinamide nucleotide adenylyl transferase 3 (NMNAT3), thus increasing its enzymatic activity, which increases NAD^+^ production and the protective effects of SIRT3. Consequently, decreased SIRT3 activity induced by cardiac pathology increases NMNAT3 acetylation, which decreases cellular NAD^+^ levels and further contributes to SIRTs’ inactivation and acceleration of cardiac dysfunction.

On the other hand, oxidative stress contributes to maladaptive cardiac remodeling: hypertrophy, heart failure, excess extracellular matrix deposition and vascular dysfunction [126,142]. Two studies have shown that SIRT3 blocks cardiac hypertrophy, both in vivo and in vitro, by decreasing oxidative damage, activating FOXO3a-dependent antioxidant-coding genes (catalase, MnSOD) and decreasing ROS levels, which ultimately suppress the activity of transcription factors involved in cardiac hypertrophy [124,136].

In the nucleus, SIRT3 acts by deacetylating and inactivating nuclear poly(ADP-ribose) polymerase, but also negatively regulates hypertrophic gene expression (brain natriuretic peptide and atrial natriuretic factor) [143]. SIRT3 deacetylates histone H3 at specific lysine residues and inhibits FOS transcription, thus decreasing the pro-inflammatory FOS-AP-1 signaling pathway [135]. Moreover, SIRT3 deacetylates FOXO1, which translocates to the nucleus and increases autophagy as a protective mechanism to reduce cardiac hypertrophy [144].

By deacetylating cyclophilin D, a regulator of the mitochondrial permeability transition pore, SIRT3 prevents age-associated increases in mitochondrial permeability and swelling, cardiac hypertrophy and fibrosis [145]. Furthermore, myocardial biopsies from obese patients with left ventricular heart failure show a 46% decrease in SIRT3 expression compared to non-obese controls, the body mass index being correlated with increased protein acetylation [146].

Overall, cardiac SIRT3 is necessary to maintain physiological mitochondrial energetics, ventricular geometry, cardiac function and coronary angiogenesis.

## 4. SIRT3 in Age-Related Neurodegenerative Diseases

SIRT3 is highly expressed in the nervous system, where it is vital for physiological neuronal processes by regulating critical brain functions [23,147,148]. Neurons are highly energy-demanding cells, as reflected by their high oxygen consumption. Mitochondria are the main source of endogenous ROS, which can induce oxidative stress, mtDNA damage and neuronal impairment [149]. Accordingly, mitochondrial malfunction plays a significant role in neurodegenerative diseases [150,151]. As neurons age, SIRT3 levels decrease and mitochondrial function deteriorates, promoting neurodegeneration [152,153,154]. Thus, SIRT3 expression decreases in the frontal cortex and hippocampi of aged rats and is accompanied by increased superoxide levels and reduced MnSOD levels [155].

In addition to the above, neurons are sensitive to NAD^+^ exhaustion. An age-related or disease-induced decline of NAD^+^ contributes to mitochondrial dysfunction and neurodegeneration by an indirect reduction in SIRTs activity [156]. NAD^+^-dependent enzymes rely on a restricted NAD^+^ pool, thus limiting each other’s activity [157]. *Per se*, increased NAD+ levels or SIRTs activity reduce the progression of neurodegeneration.

### 4.1. Age-Related Hearing Loss

In aging mammals, decreasing NAD^+^ levels contribute to lower SIRT3 activity. Moreover, humans are evolutionarily predisposed to a decrease in cochlear SIRT3 activity with age, which accelerates the decline of auditory neurons and hearing by lowering the transcription of protective factors (FOXO1, MnSOD, Hif1α), finally leading to mitochondrial dysfunction, oxidative damage and age-related hearing loss [150]. On the same note, oxidative stress contributes to age-related hearing loss. The downregulation of SIRT3 expression results in abnormal vestibular and cochlear epithelium morphology and inner ear neuron cell loss, cumulating in age-related hearing loss due to mitochondrial dysfunction and oxidative damage [158].

**Figure 2:**
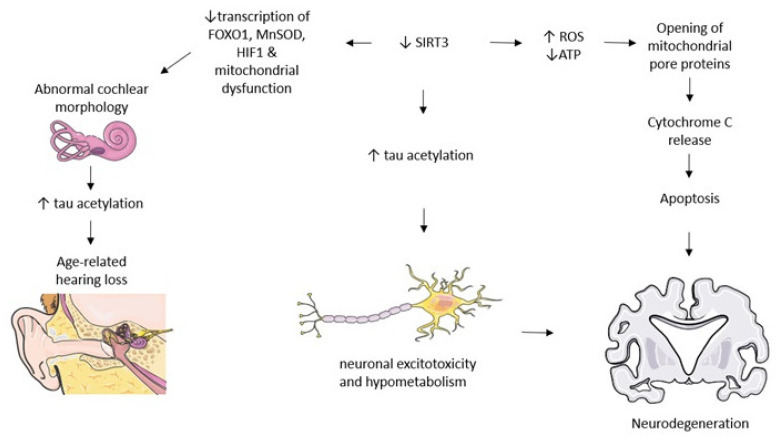
SIRT3 modulates signaling pathways relevant to neurodegenerative pathology. Decreases in SIRT3 lead to multiple physiopathological modifications that culminate with neuronal dysfunction. Abbreviations: ↑ increases, ↓ decreases.

Someya et al. [159] found that caloric restriction delays age-related hearing loss by reducing DNA oxidative lesions, the loss of spiral ganglion hair cells and neurons, but also by increasing glutathione levels and the mitochondrial antioxidation status in the cochlea. These SIRT3-dependent beneficial changes result from SIRT3 deacetylating mitochondrial isocitrate dehydrogenase 2, which increases the levels of nicotinamide adenine dinucleotide phosphate and reduces glutathione, leading to decreased oxidative stress and protection against cochlear degeneration and age-related hearing loss, as is depicted in Figure 2.

### 4.2. Alzheimer’s Disease

Restoring or maintaining mitochondrial function is central to the prevention and treatment of Alzheimer’s disease (AD) [160]. Studies have proven that dysfunctional mitochondria and dysfunctional metabolism develop early on in AD and contribute to its development [161,162,163].

Mitochondrial ROS can regulate signaling pathways relevant to neurodegeneration [164]. The first hallmark for AD diagnosis is the extracellular plaque deposits of the amyloid-β peptide. Amyloid-β accumulates in synaptic mitochondria, disturbs the mitochondrial membrane potential, increases ROS, promotes mitochondrial dysfunction [165] and decreases ATP production [166], finally leading to neuronal dysfunction and cognitive decline [167,168]. These pathological changes clearly highlight the involvement of oxidative damage in AD pathogenesis [169]. SIRT3 downregulation is associated with mitochondrial dysfunction in AD [170]. Animal and human data show that SIRT3 levels decrease as AD progresses [170,171]. Moreover, mitochondrial dysfunction leads to higher ROS levels that can upregulate amyloid-β production and deposition [172,173,174].

The accumulation of microtubule binding protein tau is the second hallmark for AD diagnosis [175]. The acetylated tau has decreased stability, promoting tau aggregation, but tau deacetylation decreases total tau levels, ameliorates tau-induced memory dysfunction and averts hippocampal atrophy [176]. Increased cerebral tau acetylation is highlighted in all the AD stages and accentuates tau accumulation and its toxicity [176,177]. It has also been shown that SIRT3 regulates tau acetylation. In the postmortem AD brains of humans and mice, SIRT3 levels are inversely correlated with tau protein and amyloid plaques, and decreased SIRT3 is associated with low cognition and severe tau pathology [178]. In mouse hippocampal cell lines, SIRT3 overexpression significantly reduces tau acetylation, while SIRT3 KO increases it [178].

Amyloid-β triggers tau pathology in the neocortex, where SIRT3 can mediate both the amyloid-β and tau pathophysiology. In an AD mouse model of amyloid-β overproduction, cortical SIRT3 mRNA and protein levels are reduced [179]. Moreover, decreased SIRT3 deacetylase activity leads to increased tau acetylation in AD brains and modulates premature tau buildup [176,180]. In human AD brains, SIRT3 levels are downregulated in the hippocampi and in the entorhinal cortex [170] compared to controls [178]. SIRT3 and its up-stream activator, PACAP, are also reduced compared to cognitively un-impaired subjects [181]. Considering the mounting evidence, the lack of SIRT3-mediated deacetylation leads to high tau acetylation and promotes its toxicity, as presented in Figure 2.

Adult neurogenesis is central to hippocampal learning and memory. It is confirmed that neural stem cells (NSCs) generate new hippocampal neurons, a vital substrate for memory [182]. Post mortem brain data show that AD patient brains have malfunctional neurogenesis accounting for their cognitive deficits [183,184]. Therefore, preserving NSCs and stimulating neurogenesis could counteract neurodegenerative diseases. In NSCs, amyloid-β is co-localized with mitochondria, where it reduces ATP levels and increases mtROS by decreasing both SIRT3 and SOD2 mRNA [185]. In addition, mitochondrial mass, biogenesis and disturbed mitochondrial dynamics consequently lead to decreased NSC proliferation, survival and differentiation. The effects of upregulating SIRT3 have not been explored in this model to date.

The neuronal network hyperexcitability of susceptible brain regions appears early in AD, predating the cognitive impairment [186,187], while animal AD models manifest neuronal circuit hyperexcitability in the cerebral cortex and hippocampus [188]. SIRT3 protects GABAergic (gamma-Aminobutyric acid) interneurons, which degenerate early on during AD, against excitotoxic DNA damage. Accumulating amyloid-β predisposes neurons to excitotoxicity [189,190] and mitochondrial dysfunction [191]. Therefore, SIRT3 reduction contributes to interneuronal vulnerability and hyperexcitability, while increasing SIRT3 expression counteracts those effects in AD.

Ion transporting ATP-ases are among the principal ATP consumers in neurons, where decreased mitochondrial ATP generation increases neuronal susceptibility to excitotoxicity [192,193]. The underlying pathway is that SIRT3 deacetylates mitochondrial proteins, thus changing their function and increasing ATP production, decreasing oxidative stress and stabilizing mitochondrial membranes and neuronal calcium transport.

Several interventional studies have demonstrated the involvement of SIRT3 in age-related neurodegenerative diseases. The most relevant studies are summarized in Table 3 [186,187,188,189,190,191,192,193,194,195].

A different pathway of SIRT3 is related to apolipoprotein E4 (ApoE4), one of the major genetic risk factors for AD [196]. In AD mice models, the ApoE4 carriers show a reduced brain glucose metabolism, ATP levels and cerebral energy generation, resulting in deficient memory and learning [204,205,206,207,208,209]. ApoE4 transgenic mice with increased SIRT3 expression are neuro-protected against hypometabolism induced by amyloid-β and have enhanced energy production [39,210].

As mentioned in previous sections, PGC-1α, a master regulator of mitochondrial biogenesis and transcriptional coactivator that modulates the expression of energy metabolism genes, transcriptionally activates SIRT3 expression. Fasting and exercise increase SIRT3 and PGC-1α, consequently enhancing mitochondrial function [211,212]. PGC-1α expression decreases proportionally with dementia progression in AD postmortem cortices. In monkeys and cultured AD mouse neurons, decreased PGC-1α was accompanied by an increased accumulation of amyloidogenic peptides and tau proteins [213,214]. Compared to their ApoE3 counterparts, ApoE4 transgenic mice show decreased PGC-1α levels in their temporal lobes, a reduced NAD^+^/NADH ratio and ATP levels [215]. ApoE2 transgenic mouse brains have an active PPAR-γ/PGC−1α signaling that is inhibited in ApoE4 ones, but PGC-1α overexpression improves the ApoE4-induced decreases in mitochondrial respiration and glycolysis [216], and also increases SIRT3 expression [57]. ApoE4 mice show decreased NAD^+^/NADH ratios and low cortical ATP levels [217,218], suggesting that ApoE4 could compromise the SIRT function by decreasing the NAD^+^ levels, mitochondrial function and ATP production [219].

ApoE4 and ApoE3 mouse cortical neurons with SIRT3 KO have reduced O2 consumption rates; conversely, SIRT3 overexpression improves O2 consumption in ApoE4 neurons. SIRT3 KO in ApoE4 neurons decreases ATP levels, whereas SIRT3 overexpression increases them, indicating that ApoE4 modulates energy metabolism through SIRT3 [215]. Thus, ApoE4 decreases mitochondrial biogenesis and induces oxidative stress and synaptic damage, finally leading to cognitive deficits, but SIRT3 overexpression counters the damaging effects of ApoE4 in mice. Therefore, the ApoE4-PGC-1α-SIRT3 may be one of the critical therapeutic targets in AD.

### 4.3. Parkinson’s Disease

Parkinson’s disease (PD) is the second most common neurodegenerative disorder worldwide, with age being its principal risk factor [220]. Dopaminergic neurons degeneration in the substantia nigra pars compacta (SNc) accounts for the main PD motor symptoms (e.g., rigidity and bradykinesia). It has been demonstrated that inflammation and ROS contribute to PD pathogenesis [221,222]. Thus, the SNc of patients with PD shows dysfunctional mitochondria and increased ROS [223,224]. Age-dependent increases in mitochondrial oxidative stress also contribute to SNc dopaminergic neuron degeneration [225]. Additionally, hereditary early onset PD forms are caused by dysfunctional genes regulating mitochondrial anti-oxidation [226] and mitochondrial quality control (mutations altering the PINK1 kinase domain) [227].

SIRT3 protects dopaminergic neurons in the SNc against age-dependent increases in mitochondrial malfunction and oxidative stress. Consequently, the loss of SIRT3 decreases the antioxidant activity and increases oxidative stress. Shi et al. [228] found that MnSOD is significantly more acetylated in the SNc from patients with PD than in that from controls. One of the principal anti-oxidant mechanisms is SIRT3 deacetylating MnSOD in SNc dopaminergic neurons, thus increasing its anti-oxidant activity [228].

In a rotenone-induced PD model, SIRT3 upregulation decreases apoptosis and ROS levels, prevents alpha-synuclein accumulation, ameliorates glutathione and SOD levels and enhances cell viability [229]. Furthermore, in in vitro and in vivo PD models, miR-494-3p (a specific miRNAs) negatively regulates SIRT3. In this respect, Geng et al. [230] found that inhibiting miR-494-3p leads to the upregulation of SIRT3 and ameliorates the PD phenotype.

Regarding cell energy pathways, SIRT3 deacetylates and activates citric acid cycle-ATP producing enzymes (pyruvate dehydrogenase, citrate synthetase) that are downregulated by age-related diseases [231]. In vitro PD models show reduced enzymatic activity and levels of SIRT3, isocitrate dehydrogenase and citrate synthetase levels. Thus, SIRT3 overexpression restores citrate synthetase activity, decreases its acetylation and partially reverses ATP depletion, but has no effect on pyruvate dehydrogenase and isocitrate dehydrogenase 2 [231].

Knowing that HT is a risk factor for PD, antihypertensive agents protect against PD, but the role of SIRT3 in humans is unknown in this regard [232]. In aged rats, ANGII decreases SNc SIRT3 levels and may contribute to neurodegeneration, an effect counteracted by ANGII antagonists [233]. Whether this also applies to humans has not been established to date. Therefore, targeting indirect SIRT3 inhibitors, such as angiotensin 1, with its antagonists that are used as hypertensives, may ameliorate PD [233].

## 5. SIRT3 Regulates Interventions That Enhance Health and Lifespan

### 5.1. Fasting

Murine and human data show that fasting counteracts age-related diseases such as neurodegenerative, cancer and cardiovascular disease [234,235]. Nutritional input strongly influences the mitochondrial acetylome and SIRT activity. Fasting induces a catabolic state that generates acetyl-CoA from fatty deposits that increase the acetylation levels of mitochondrial proteins [36,236]. Dietary restriction in mice induces hepatic SIRT3 expression that reversibly deacetylates mitochondrial enzymes and contributes to fatty acid oxidation [34], ketone body generation [24] and usage [237]. Moreover, SIRT3 activity is upregulated during fasting to combat hyperacetylation [34].

Long-term cognitive and behavioral adaptation to fasting requires hippocampal SIRT3. Rodent models show that fasting has a neuroprotective effect in stroke [238] and PD [239] by upregulating antioxidant and neurotrophic factor expression, suppressing inflammation [240] and upregulating GABA synaptic signaling in an SIRT3-dependent fashion, consequently decreasing neuronal excitability [241].

Low levels of mitochondrial ROS partake in physiological cellular signaling, but high levels that persist over longer periods cause neuronal degeneration and synaptic dysfunction [242,243]. During fasting, SOD2 deficiency prevents adaptive increases in synaptic activity inhibition. It was proven that SIRT3 levels increase two-fold in hippocampal cells of mice adapted to fasting, that SIRT3 is necessary for maintaining synaptic plasticity and hippocampal memory, and also that SIRT3 deacetylates and activates SOD2, thus regulating the activity of hippocampal neuronal networks during fasting [241].

Human and animal data show that neuronal hyperexcitability arises early in AD, before neuronal degeneration of affected neural networks [244,245,246], due to the decreased GABA synaptic activity of the hyperexcitable neuronal networks [247,248]. In AD mouse models, fasting suppresses seizure development, ameliorates memory deficits and spatial learning, and increases hippocampal synaptic plasticity. Thus, fasting activates SIRT3, which deacetylates SOD2, leading to a decrease in ROS and blunted inflammasome activation [117].

SIRT3 upregulates GABAergic tone in mouse hippocampi adjusted to an intermittent fasting schedule [241]. Therefore, AD neuronal network malfunction could be reversed via pharmacological and dietary interventions that promote GABAergic interneuronal functionality.

Considering all the studies mentioned above, we can conclude that SIRT3 ensures metabolic plasticity in nutrient deficient conditions and intermittent fasting can improve cognition and hippocampal plasticity via SIRT3-regulated decreases in hyperexcitability.

### 5.2. Physical Exercise

Aging results in muscle mass loss (sarcopenia), decreased mitochondrial respiration, lower SIRT3 and PGC-1α levels and decreased muscle performance [249], which promotes increases frailty and morbidity [250]. Aging individuals are also less physically active; sedentarism induces muscle atrophy and reduces mitochondrial mass and biogenesis regulators (SIRT3 and PGC-1α) in human muscle cells, which further predisposes the individuals to sarcopenia.

Physical exercise reverses these changes in elderly subjects [251]. Life-long exercising elderly subjects have increased muscle SIRT3 and SOD levels [252] that counter age-associated oxidative stress and mitochondrial deterioration [253]. Elderly individuals who exercise have a youth-like gene expression profile (increased SIRT1, SIRT3, catalase, SOD1) and improved cognitive functions compared to age-matched sedentary controls [252,254]. Therefore, physical exercise deters the damaging effects of aging and protects against age-associated diseases [255,256].

Regarding age-related neurological degradation, it has been proven that aerobic exercise induces cortical and hippocampal SIRT3 expression [19]. The mechanism lying behind this is that hippocampal neurons upregulate SIRT3 as a response to exercise, thus protecting neurons and mitochondria against metabolic stress [19].

Compared to young controls, aged sedentary rats show a decrease in mARN levels of SIRT3, SIRT1, insulin-like growth factor 1 and vascular endothelial growth factor [257]. In that study, treadmill running significantly increased those biomolecules and inhibited the pro-inflammatory state that predisposes dopaminergic neurons to PD with aging.

### 5.3. Exogenous Ketones

Ketones are neuroprotective agents [258,259]. Ketogenic diets are useful in treating multiple neurodegenerative diseases, such as drug-resistant epilepsy [260], AD [261] and PD [262]. It has also been proven that ketogenic diets reduce mortality and aging, and improve memory in aged mice [263]. As a mechanism of action, ketogenic diets can increase mitochondrial biogenesis and efficiency in neurons [264,265,266], consequently increasing ATP levels and improving neuronal energy metabolism, thus combating neurodegeneration [267].

ApoE4-positive individuals have cerebral hypometabolism. Amyloid-β is associated with downregulated SIRT3, consequently promoting neuronal hypometabolism and AD progression [178,210]. The administration of exogenous ketones decreases amyloid entry into neurons and improves learning and memory in Alzheimer mouse models [258].

Compared to ApoE3 mice, ApoE4 mice show impaired learning and memory. ApoE4 mice treated with exogenous ketones have improved memory and learning compared to controls. Exogenous ketones increase ATP levels and the NAD^+^/NADH ratio in ApoE4 mouse cortical tissue and hippocampi. Three months of exogenous ketones increase SIRT3, post synaptic density protein 95 and synaptophysin in the hippocampi and cortices of ApoE4 mice but not in ApoE3 mice [39]. Overall, exogenous ketones increase SIRT3 expression and the NAD^+^/NADH ratio and improve synaptic integrity, learning and memory in ApoE4 mice. Mechanistically, exogenous ketones may act via increasing the NAD^+^/NADH ratio, thus increasing the SIRT3-required coenzyme and its activity and increasing mitochondrial ATP production.

Hasan-Olive et al. [268] showed that oxidative stress decreases the mitochondrial activity and cellular levels of PGC-1α in human fibroblast cell lines, but beta-hydroxy butyrate salvages mitochondrial activity from oxidative stress-induced dysfunction. In cultured fibroblasts and hippocampal mouse neurons, exogenous beta-hydroxy butyrate and ketogenic diets increase PGC-1α levels and mitochondrial biogenesis compared to standard diet-fed rats by increasing SIRT3 levels [268].

On the same note, ketone esters reduce behavioral deficits in AD mouse models [269]. In Sirt3+/− AppPs1 mice, chronic ketone esters’ administration increases cerebral SIRT3 and decreases interneuron death. Ketone esters protect GABAergic interneurons and prevent hyperexcitability; hence, by congruence, ketone precursors may be beneficial in AD [270]. Beta-hydroxy butyrate protects GABAergic interneurons against an amyloid-β-induced degeneration via SIRT3 activity. Thus, mitochondria may mediate the beneficial effects of ketones in AD and cognition [269]. Consequently, increasing mitochondrial NAD^+^ levels via NAD^+^ precursors enhances SIRT3 activity and protects neurons against oxidative stress and amyloid-β [197,271,272].

## 6. Conclusions

SIRT3 regulates intra and extra-mitochondrial proteins to maintain cell function. SIRT3 activity is central to mitochondrial and cellular homeostasis throughout the life of an organism. Stimulating SIRT3 activity can delay the progression of cellular hallmarks of aging and hinder the development of age-associated diseases. By regulating the mitochondrial acetylome, SIRT3 reduces excess ROS, maintains the physiological activity of diverse cellular signaling pathways and protects against disease. Further research is warranted to elucidate all the molecular mechanisms mediating the protective effects of SIRT3 and how post-translational modifications affect SIRT3 activity. Translating pre-clinical findings into applicable interventions to humans requires the additional development of molecules that directly or indirectly increase SIRT3 activity. Overall, activating SIRT3 is a viable therapeutic strategy to improve human health and lifespan. Therefore, obtaining effective mitochondria-targeted treatments is a goal worth pursuing.

## Figures and Tables

**Table 1:** Interventions that modulate SIRT3 signaling in hypertension.

Experimental Model	Intervention	The Effect of Intervention	Reference
Wild type mice	Angiotensin II induced hypertensive renal nephropathy	Decreased kidney SIRT3 expression; HT; Decreased renal function;	[120]
SIRT3 KO mice	Angiotensin II induced hypertensive renal nephropathy	Hypertensive nephropathy, renal fibrosis;	
Wild type mice	Angiotensin II induced hypertensive renal nephropathy + Honokiol (bioactive compound from *Magnolia officinalis*)	↑ Renal function; ↑ SIRT3 expression; ↓ Kidney fibrosis; Activates SIRT3-KLF15 signaling pathway.	
Male newborn rats	Short-term sucrose ingestion in the early post-natal period	↑ Blood pressure; ↓ Aortic SIRT3, SOD2 and endothelial NO synthase expression; ↑ Energy supply, ↓NADH to NAD^+^ oxidation; ↓SIRT3 activity.	[121]
Spontaneously hypertensive rats	α-Linolenic acid	↓ HT; ↑ Endothelial function; Prevents HT-induced SIRT3 reduction and SOD2 hyperacetylation; ↓ Endothelial mtROS; Improvements were SIRT3-dependent.	[122]
Human umbilical vein endothelial cells	Resveratrol	↓ Oxidative damage, mtROS, apoptosis; ↑ Cell viability; Activates AMPK-PGC-1*α* signaling, ↑ SIRT3 transcription, ↑ deacetylation+activation of mtROS clearing enzymes, ↑ complex 1 activity and ATP synthesis.	[123]

Abbreviations: ↑ increases, ↓ decreases.

**Table 2:** Interventions that modulate SIRT3 signaling in cardiac hypertrophy.

Experimental Model	Intervention	The Effect of Intervention	Reference
Adult male CD-1 mice with induced cardiac fibrosis	Honokiol	Protects against the appearance and progression of cardiac hypertrophy by activating mitochondrial SIRT3; Honokiol directly binds to SIRT3 and increases its enzymatic activity, affinity for NAD^+^ and gene expression.	[124]
Male Sprague Dawley rats; Transverse aortic constriction (TAC)-induced hypertrophy	Choline	Decreases cardiac hypertrophy and fibroses by activating SIRT3-AMPK-UPRmt signaling; Improves metabolic function; Increases serum beta-hydroxy butyrate and acetylcholine levels; Increases cardiac levels of enzymes required to metabolize ketone bodies and fatty acids that decrease cardiac hypertrophy.	[125]
TAC mice models	Sesamin	Decreases cardiac hypertrophy, fibrosis and inflammation; Improves cardiac function; Sesamin-induced reduction in hypertrophy is dependent on SIRT3, which decreases ROS; Increases SIRT3 and SOD2 expression and decreases FOXO3a phosphorylation.	[126]
In vitro neonatal rat cardiomyocytes hypertrophy model induced by ANGII	Hydrogen sulfide	↑ SIRT3 promoter activity and expression; ↓ Hypertrophy, ↑ mt function, ↑ SOD2 and FOXO3a expression, ↓ oxidative stress; All hydrogen sulfide-induced changes were SIRT3-dependent.	[127]
Mice with TAC induced hypertrophy		↓ Cardiac hypertrophy, ↓ ROS, ↓ Blood pressure; Restores myocardial mitochondrial structure, number and volume; ↑ OPA1, MFN1, MFN2 (mitochondrial fusion genes that increase respiratory chain efficiency) and pro PGC-1α, all the modifications being SIRT3 dependent.	[127]
Mice with TAC induced hypertrophy	Dihydromyricetin	↓ Hypertrophy, ↓ ROS, ↑ expression and activity of SIRT3, FOXO3a, SOD2; Activates AMPK-PGC1alpha-ERRalpha axis, which increases SIRT3 expression and leads to mtSOD2 deacetylation and decreased oxidative damage.	[128] [129]
	Resveratrol	↓ Cardiac hypertrophy and collagen deposition, ↑ Cardiac function, all in an SIRT3-dependent manner; In vitro, prevents fibroblast-myoblast differentiation by inhibiting TGFbeta-Smad3 signaling.	[130]
	NAD^+^	↓ Hypertrophy in a SIRT3-dependent manner by activating SIRT3-LKB1-AMPK signaling and culminates with decreased mTOR activity and decreased hypertrophy; Pathologic cardiac hypertrophy decreases Nampt and NAD^+^ levels (but not in exercise-induced hypertrophy).	[131]
	Emodin	↑ PGC-1α-SIRT3 signaling.	[132]
Angiotensin II induced hypertrophy in cardiomyoblast H9c2 cells	1,25-OH vitamin D3	↓ Hypertrophy in a SIRT3 independent manner; SIRT3 expression was unaffected by the intervention.	[133]

Abbreviations: ↑ increases, ↓ decreases.

**Table 3:** Interventions that modulate SIRT3 signaling in age-related neurodegenerative diseases.

Experimental Model	Intervention	The Effect of Intervention	Reference
In vitro: amyloid β oligomer-treated primary hippocampal neuronal cells In vivo: transgenic PS1V97L mouse model	Honokiol	Ameliorates mitochondrial dysfunction by activating SIRT3 and increasing its levels, which results in suppressed ROS, an increased ATP production, normalized mitochondrial membrane potential, delayed cognitive impairment; Decreases amyloid-β-induced hippocampal neuron apoptosis and improves cognitive performance.	[194] [195] [196]
DNA repair deficiency mouse (3xTgAD/Polβ^+/−^)	Nicotinamide riboside	Improves memory, learning and motor function; Decreases systemic inflammation, phosphorylated tau, DNA damage and apoptosis; Restores SIRT3 and SIRT6 levels; Restores synaptic plasticity in the hippocampus; Increases deacetylated SOD2 and increases neurogenesis; No effect on amyloid-β production.	[197]
In vitro and in vivo gentamicin-induced hair cell loss model	Adjudin	Protects against gentamicin-induced hair cell loss in rats’ cochleae by increasing SIRT3 mRNA and protein levels expression and decreasing ROS.	[198]
Noise-induced hearing loss mouse models	Nicotinamide riboside	Protects against degeneration of spiral ganglion neurites and noise-induced hearing loss in a SIRT3-dependent manner; Increases mitochondrial NAD^+^ and SIRT3 activity.	[199]
Four-week-old Sprague Dawley rats	d-Galactose-induced aging	Decreases SIRT3 expression, mtDNA lesions and SOD2 activity; Increases malondialdehyde and apoptosis levels in rats’ auditory cortices in natural and D-galactose-induced aging.	[200]
Peripheral lymphocytes from patients with AD	Resveratrol	AD patient lymphocytes show increased oxidative stress. Selenium administration did not modify the expression of SIRT3 or other longevity-related genes, but resveratrol upregulated SIRT3, SIRT1, SOD2 and NRF2 (a transcription factor that activates antioxidant response genes), that could be responsible for SIRT3 upregulation after resveratrol administration and provide protective effects in AD afflicted cells.	[201] [202] [203]

## Abbreviations

AD	Alzheimer’s disease
AMPK	AMP-activated protein kinase
ANGII	Angiotensin II
APOE	Apolipoprotein E
ATP	Adenosine triphosphate
CVD	Cardiovascular diseases
FOXO3A	Forkhead box O3
GABA	Gamma-Aminobutyric acid
GSK3β	Glycogen synthase kinase 3 beta
HIF1alpha	Hypoxia-inducible factor 1-alpha
HT	Hypertension
KO	Knock-out
MCP-1	Monocyte chemoattractant protein 1
MnSOD	Manganese superoxide dismutase
mRNA	Messenger RNA
mtROS	Mitochondrial ROS
mtDNA	Mitochondrial DNA
NAD^+^	Nicotinamide adenine dinucleotide
NFkB	Nuclear factor kappa-light-chain-enhancer of activated B cells
NLRP3	NOD-, LRR- and pyrin domain-containing protein 3
NMNAT3	Nicotinamide nucleotide adenylyl transferase 3
NO	Nitric oxide
NSC	Neural stem cells
OPA1	Optic atrophy 1
PD	Parkinson’s disease
PGC-1α	Peroxisome proliferator-activated receptor-gamma coactivator
PPARα	Peroxisome proliferator-activated receptor
ROS	Reactive oxygen species
SIRTs	Sirtuins
SIRT3	Sirtuin 3
SNc	Substantia nigra pars compacta
SOD	Superoxide dismutase
TAC	Transverse aortic constriction
TGF-β	Transforming growth factor β
VCAM-1	Vascular cell adhesion molecule 1
